# Benefits of Docosahexaenoic Acid, Folic Acid, Vitamin D and Iodine on Foetal and Infant Brain Development and Function Following Maternal Supplementation during Pregnancy and Lactation

**DOI:** 10.3390/nu4070799

**Published:** 2012-07-24

**Authors:** Nancy L. Morse

**Affiliations:** Efamol Ltd., 14 Mole Business Park, Leatherhead KT22 7BA, UK; Email: nancy.morse@wassen.com; Tel.: +1-902-538-8762; Fax: +1-902-538-1443

**Keywords:** docosahexaenoic acid, DHA, vitamin D, folic acid, iodine, foetal development, infant development, brain function, brain development, eye function

## Abstract

Scientific literature is increasingly reporting on dietary deficiencies in many populations of some nutrients critical for foetal and infant brain development and function. Purpose: To highlight the potential benefits of maternal supplementation with docosahexaenoic acid (DHA) and other important complimentary nutrients, including vitamin D, folic acid and iodine during pregnancy and/or breast feeding for foetal and/or infant brain development and/or function. Methods: English language systematic reviews, meta-analyses, randomised controlled trials, cohort studies, cross-sectional and case-control studies were obtained through searches on MEDLINE and the Cochrane Register of Controlled Trials from January 2000 through to February 2012 and reference lists of retrieved articles. Reports were selected if they included benefits and harms of maternal supplementation of DHA, vitamin D, folic acid or iodine supplementation during pregnancy and/or lactation. Results: Maternal DHA intake during pregnancy and/or lactation can prolong high risk pregnancies, increase birth weight, head circumference and birth length, and can enhance visual acuity, hand and eye co-ordination, attention, problem solving and information processing. Vitamin D helps maintain pregnancy and promotes normal skeletal and brain development. Folic acid is necessary for normal foetal spine, brain and skull development. Iodine is essential for thyroid hormone production necessary for normal brain and nervous system development during gestation that impacts childhood function. Conclusion: Maternal supplementation within recommended safe intakes in populations with dietary deficiencies may prevent many brain and central nervous system malfunctions and even enhance brain development and function in their offspring.

## 1. Introduction

The foetus and breastfed infant is totally dependent on maternal nutrient status for growth and development. Recent research has shown that maternal dietary deficiencies of docosahexaenoic acid (DHA), vitamin D, folic acid and iodine are associated with a variety of poor foetal and/or infant health outcomes mostly impacting brain development and/or function in infancy and often throughout life. Therefore, adequate maternal nutrient intake is critical when planning to conceive and during pregnancy and lactation.

A review of current literature was undertaken to summarize the potential benefits of maternal supplementation with DHA, vitamin D, folic acid and iodine during pregnancy and/or breast feeding for foetal and/or infant brain development and/or function. A systematic search was performed in MEDLINE for English-language articles published between January 2000 and February 2012 using broad search criteria including *DHA and pregnancy*, *DHA and lactation*, *docosahexaenoic acid and pregnancy*, *docosahexaenoic acid and lactation*, *vitamin D and pregnancy*, *vitamin D and lactation*, *folic acid and pregnancy*, *folic acid and lactation*, *iodine and pregnancy* and *iodine and lactation*. Additional studies including some prior to January 2000 were identified within the Cochrane Central Register of Controlled Trials and by reviewing reference lists from included studies and review articles. Titles and abstracts were reviewed and reports were selected for inclusion in the review if they were systematic reviews, meta-analyses, randomised controlled trials, cohort studies, cross-sectional or case-control studies and if they reported benefits and/or harms associated with maternal supplementation with DHA, vitamin D, folic acid or iodine during pregnancy and/or lactation. Studies that reported neither benefit nor harm were not included.

Data was reviewed and summarized to discuss the relevance of dietary DHA, vitamin D, folic acid and iodine to foetal and infant brain development and function, to present evidence demonstrating dietary deficiency of these nutrients in many populations, to highlight the potential benefits of maternal supplementation during pregnancy and/or lactation on foetal and/or infant outcomes and to include safe intake recommendations. 

### 1.1. DHA

Over the past three decades our diets have changed enormously. We have been encouraged to reduce fat intake while at the same time detrimental *trans* fatty acids have been introduced into the food chain. In response, many people have reduced intake of *all* dietary fat without realizing that there is a requirement for certain fats especially for women during pregnancy and while breast feeding, in particular the omega-3 fatty acid, docosahexanoic acid (DHA). 

Clinically established as a nutrient essential for the development of an infant’s brain and central nervous system, DHA occurs naturally in breast milk, and is added to infant formula [1]. In the last trimester of pregnancy, the foetal brain increases in size while rapidly accumulating DHA [2]. As reported in this review, foetal and infant DHA deficiencies are associated with poor growth, and brain and eye development and function. Numerous observational studies have identified a link between maternal DHA intake during pregnancy and while breast feeding, and enhanced foetal and infant development and function. In addition, intervention trials have measured significant benefits for both the mother and baby. 

#### 1.1.1. Importance of Fatty Acids in Brain Development and Function

Fatty acids such as DHA are found in dietary fat and are components of every cell membrane in the body. The types of fatty acids in the diet influence body composition, and ultimately its function and health. 

Fatty acids are grouped into various categories: for example saturated fatty acids tend to be solid at room temperature and are abundant in butter. Polyunsaturated fatty acids (PUFAs) are liquid at room temperature and are the main components of vegetable oils such as corn, sesame and evening primrose, and are also found in fish and fish oils. PUFAs are often called “good fats” because eating a higher proportion of them compared to saturated fats can improve health. These are subdivided into two main categories, omega-6 and omega-3. Various long chain polyunsaturated fatty acids (LC-PUFAs) within these two categories can be synthesized de novo starting with dietary essential fatty acids (EFAs), the omega-6 linoleic acid (LA) and the omega-3 alpha-linolenic acid (ALA) respectively, through a multi-step process that is very slow and inefficient in humans [3,4]. Typically, only about 0.1% of dietary ALA is converted to DHA in normal healthy adults eating a Westernized diet [5], making routine dietary intake of DHA a necessity in extraordinary circumstances, such as in pregnancy and during lactation. 

About 60% of the dry weight of brain tissue is fat. The most abundant LC-PUFAs in the brain and those which are critical for proper brain, nervous system and eye development and function are DHA and the omega-6 arachidonic acid (AA). DHA and AA are highly concentrated in membrane phospholipids of the retina and brain, where they accumulate rapidly during foetal and infant growth spurts [6,7]. DHA is the main structural fatty acid in nerve cells and its presence helps to ensure nerve cell message transmission through its effects on ion channels, response to neurotransmitters [8], and formation of secondary messengers [9]. It may also protect against loss of scaffolding proteins [10,11] and lipid peroxidation [12,13] thereby maintaining the physical structure of the brain. DHA is also extremely important for vision since it is the main membrane constituent in the photoreceptor cells of the eye. These cells are responsible for transmitting light messages to nerves that supply the brain and their proper function is essential for vision. 

#### 1.1.2. Maternal Nutrition: During Preconception, Gestation and Lactation

The parent EFAs and their derived LC-PUFAs are vitally important structural elements of all cell membranes, so they are absolutely essential for formation of new tissue as occurs throughout foetal development. During pregnancy and while breast feeding, mothers are the sole provider of these important nutrients to the growing fetus and baby. Consequently, maternal fatty acid status is critical to ensure optimal supply to the offspring, and maternal dietary intake must be sufficient to satisfy her requirements as well as those of her growing baby. 

**Figure 1:**
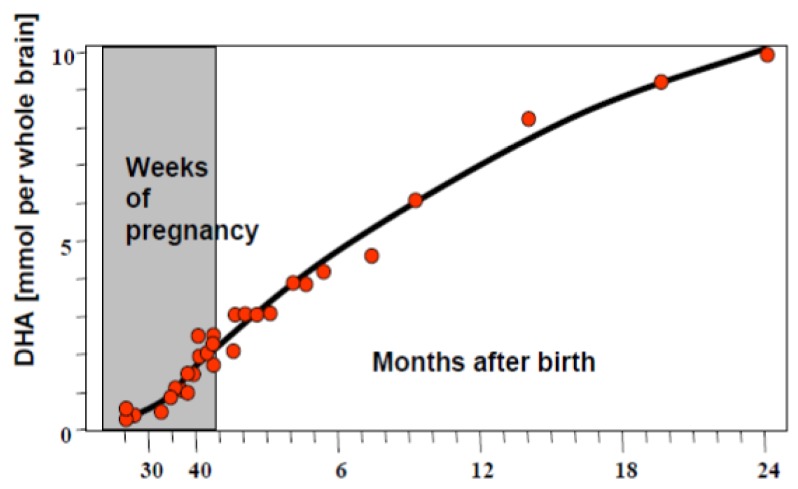
Docosahexaenoic acid (DHA) accumulation in foetal brain [2].

LC-PUFAs are required during all reproductive stages. Before pregnancy, they ensure that the mother’s body is well nourished before she conceives so that the pregnancy begins in a healthy state. During pregnancy they are required for growth of the mammary glands, placenta, uterus and fetus. In the last three months of pregnancy, there is rapid accumulation of DHA in the eyes and brain of the foetus (Figure 1) [2] and its brain weight increases, making it increasing important that the mother has an adequate DHA intake at this time. 

After birth, the baby’s nervous system continues to grow very rapidly and DHA supplied primarily through breast milk, is required as a structural component. Consequently, maternal body stores can become depleted resulting in health risks for her including post natal depression [14,15,16].

During the last trimester, a foetus accrues about 67 mg of DHA per day from the mother, and during breast feeding the need increases to 70–80 mg daily [17]. This huge demand for DHA particularly during breast feeding depletes maternal stores to below pre-pregnancy levels and this deficit can take months to even partially correct.

**Figure 2:**
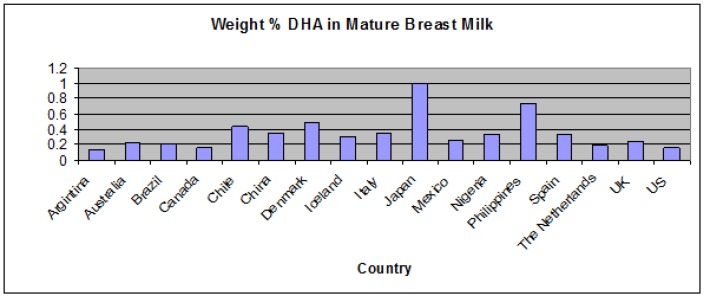
Variation in DHA content of mature breast milk obtained from mothers in various countries [18,19,20,21,22,23,24,25,26].

In addition, the LC-PUFA content of breast milk can vary widely from mother to mother depending on her diet and how efficiently she is able to make these nutrients from the parent EFAs (Figure 2).

**Figure 3:**
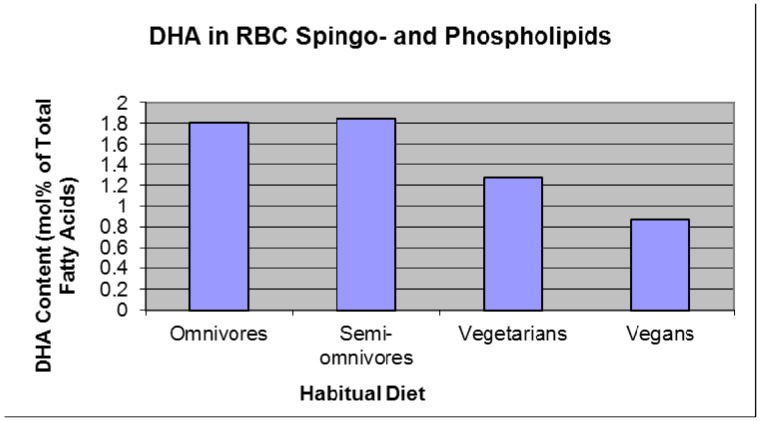
DHA status relative to dietary habit [27,28,29].

A number of dietary and environmental factors can affect the fatty acid status of the mother. Vegetarians have lower than normal DHA status (Figure 3) [27,28,29] because a strict vegetarian diet does not contain any DHA. 

**Figure 4:**
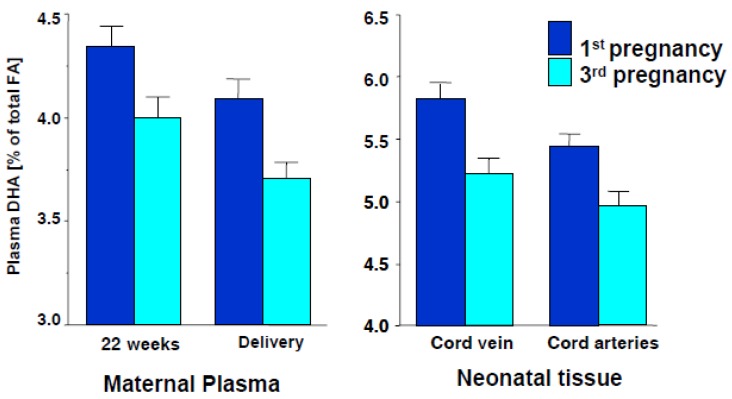
DHA status in successful pregnancies [30].

Also, mothers who have given birth in rapid succession and those who have given birth to twins, triplets or other multiples have lower than normal levels of DHA [30,31]. This was initially deduced from a population study completed at Maastricht University, The Netherlands, where the fatty acid status of 98 mothers of singletons and 146 mothers of twins, triplets or other multiples was determined during pregnancy and after delivery. During this study, the fatty acid status of their infants was also assessed immediately following birth. Results showed the infant’s DHA status was progressively lower as the number of infants per pregnancy increased and as the number of singleton births increased (*i.e.*, a first born had higher DHA levels than a second born, *etc.*). Consequently, the mother’s DHA status becomes reduced after each successive pregnancy, restricting the supply of this nutrient to the growing fetus and results in low DHA status in the infant (Figure 4). 

However, dietary supplementation can increase maternal plasma and breast milk DHA which can be passed on to the growing baby. 

#### 1.1.3. Infant Supplementation Studies

The idea that LC-PUFAs may be important for early brain development and function came from comparison studies between infants fed mother’s milk which contains LC-PUFAs and those fed formula without LC-PUFAs. These studies [32,33,34,35], plus intervention trials [36,37,38,39,40,41,42,43] that included formula supplemented with LC-PUFAs, have reported enhanced eye development and function in infants, in particular visual acuity [41], and less conclusively enhanced infant brain development and function pertaining to problem solving ability [41]. These results furnished a compelling argument that LC-PUFAs may also be important for the growing foetus. 

### 1.2. Vitamin D

Vitamin D is a fat soluble vitamin found in some foods including fish and eggs, and can also be manufactured in skin upon exposure to ultraviolet B rays from sunlight. Vitamin D is required to maintain pregnancy, for skeletal development, and to promote normal brain development. There is evidence of widespread sub-clinical vitamin D deficiency [44] that is aggravated by long hours of work indoors and avoidance of sunshine aimed at reducing skin cancer risk [45]. 

Vitamin D exists in several different forms including D1, D2, D3, D4 and D5 that differ primarily in their side chains. The two major forms are vitamin D2 or ergocalciferol, and vitamin D3 or cholecalciferol. These are known collectively as calciferol. The majority of circulating vitamin D, known as serum 25-hydroxyvitamin D [25(OH)D] that is necessary to maintain health and function of the immune, reproductive, muscular, skeletal and integumentary system, originates from vitamin D3 (cholecalciferol) and reflects endogenous synthesis from exposure to sunlight as well as intake from the diet [46]. 

There are very few dietary sources of vitamin D. Oily fish such as herring, mackerel, pilchards, sardines and tuna are rich sources but their consumption in some countries is low. The only other useful sources are eggs, fortified margarines (required in some countries by law to contain vitamin D) and some fortified yoghurts and breakfast cereals. However, a recent global review of vitamin D status has shown that its intake is often too low to sustain healthy circulating 25(OH)D in countries without mandatory staple food fortification and is even too low in countries that do fortify due to low milk consumption, vegetarianism, non-supplement use and low fish intake [46]. Supplement use contributed 6%–47% of the average vitamin D intake in some countries. As reported in 2005, the average dietary intake of vitamin D was in the range of 3 μg/day in most countries and did not exceed 9 μg/day in any of the countries surveyed including the United States, Canada, the United Kingdom, Ireland, Scotland, Australia, Europe, Japan and various other countries. 

Vitamin D deficiency is defined as serum 25(OH)D of less than 25–50 nmol/L. Approximately one billion people worldwide are estimated to be vitamin D deficient with people living in Europe, the Middle East, China and Japan at particular risk [47,48]. Deficiency is more common in women than men (9.2% *vs.* 6.6%) and pregnancy is known to represent a particularly high-risk situation [45]. In addition, pregnant women with darker skin pigmentation are at even greater risk of low vitamin D status as compared to pregnant women with lighter skin pigmentation [49].

Vitamin D is important during pregnancy to:

1. Build strong bones—vitamin D ensures foetal supply of calcium for strong bones [45] including those of the skull. Severe hypocalcaemic is associated with high risk of brain damage [50]. vitamin D insufficiency has been associated with reduction in bone mineral content of the offspring [51] and perinatal growth restriction [52].
2. Maintain pregnancy—the circulating concentration of maternal active vitamin D rises in the first trimester and doubles by the end of the third trimester [53]. The early rise is believed to be necessary to enable immunological adaptation by the mother that is required to maintain normal pregnancy [53]. These vitamin D induced immunological changes in the mother prevent miscarriage [45,53].
3. Promote normal brain development—preliminary research suggests that gestational vitamin D insufficiency has been linked to altered brain development and adult mental health [49], in particular schizophrenia [54].

There is also evidence from observational studies suggesting that adequate vitamin D during early life may prevent development of immunological diseases in the offspring later in life such as Type 1 diabetes [55], allergic diseases [53] and lower respiratory tract infections, wheezing and asthma [56]. Therefore at its worst, vitamin D deficiency can be life threatening to the newborn, while lesser deficiency can weaken skull bones risking brain injury during birth and can contribute to a multitude of future health problems.

### 1.3. Folic Acid

Folic acid is a B vitamin that plays an important role in cell division, and synthesis of amino acids and nucleic acids and is therefore essential for growth [57]. It is necessary for normal development of the foetal spine, brain and skull, in particular during the first four weeks of pregnancy.

During pregnancy the rate of cell division and erythrocyte formation increases dramatically as the uterus enlarges, the placenta develops, maternal blood volume increases and the embryo develops into a foetus [58]. In addition, folate is transferred from the mother to the growing foetus [57] increasing the demand for folate beyond her sole requirements. Women at risk of low folate status include [59,60,61,62]:

- Those not taking the recommended quantity of folic acid supplement;
- Those on restricted diets (chronic dieters);
- Those with lower socio-economic status;
- Those with limited or uncertain availability of nutritionally adequate and safe food.

Studies have reported a decreased risk of neural tube defects including malformations of the spinal column (spina bifida) and the skull (anencephaly) is associated with both increased maternal folate intake and higher maternal red blood cell folate concentration (greater than 906 nmol/L) [58]. Neural tube defects occur during the third and fourth week of pregnancy, before the woman knows she is pregnant, and involve failure of the neural tube to close properly. This risk is reduced when the mother takes a daily multivitamin containing folic acid three months before pregnancy and continuing up to the 6th week from the beginning of her last menses [63].

Considering this evidence and recognizing that pregnancies are not always planned, the requirement for folic acid in women of child bearing age and during pregnancy has become well established and internationally recognized (see Section 6.3 under Safe Intake Recommendations). Steps to achieve folate sufficiency have included mandatory or voluntary food fortification in some countries such as Canada [63] and New Zealand [64], and the promotion of folate supplementation for all women who could become pregnant. 

Even with wide spread recognition of the need for folic acid to prevent neural tube defects, it is still not widely used in the general population globally. For example, in 2008 a systematic review of relevant research from 1989 to May 2006 in Europe, the USA, Canada, Australia and New Zealand was used to make recommendations to improve folic acid supplement use in the UK, particularly among low-income and young women. It included 26 systematic reviews and/or meta-analyses identified from the wider public health literature, and 18 studies on the effectiveness of preconception interventions. The results showed that even high-quality public relations campaigns that increase use result in under half of women in the target group taking supplements [65].

### 1.4. Iodine

Iodine is an essential mineral that humans need to produce thyroid hormones throughout life. These hormones are especially needed to ensure normal development of the brain and nervous system during gestation and early life [66]. Since the foetus is totally dependent in early pregnancy on maternal thyroid hormones for normal brain development, it is very important that pregnant women consume enough iodine [67]. During lactation, the mammary glands concentrate iodine within breast milk to nourish the newborn [66] whose iodine requirement is approximately 7 μg/kg of body weight [66].

The two thyroid hormones that contain iodine are thyroxine (T4) and triiodothyronine (T3), the later being the biologically active form. T4 has four iodine molecules while T3 has three. Within the body, dietary iodine mixes with circulating iodine originating from iodine molecules removed from thyroid hormones to create a pool of inorganic iodide available for metabolic use [68]. This pool is in a dynamic equilibrium where the thyroid takes iodide that is required for T3 and T4 synthesis and the kidneys filter and excrete excess iodide in the urine [68]. 

In a healthy non-pregnant woman with adequate iodine intake, the absorbed dietary iodine balances renal iodide clearance and the thyroid maintains a normal iodine store of 15–20 mg [69]. If iodine intake is inadequate before pregnancy, maternal deficiency may result in inadequate supply of iodine for the unborn baby in later stages of pregnancy [70]. In addition, when a woman becomes pregnant, her iodine requirement increases more than 50% [69] to 220–250 μg/day [71] due to:

1. An increase in maternal T4 concentration to maintain her normal thyroid hormone levels while transferring additional thyroid hormone to the foetus early in the first trimester (before the foetal thyroid is functioning) [66];
2. Iodine transfer to the foetus, particularly towards the end of pregnancy [66];
3. An increase in iodine urinary excretion [66].

The rate of maternal thyroid hormone production returns to normal following birth. However, iodine supplementation is also recommended during breast feeding because infants are completely dependent on their food to supply iodine to build their own reserves of thyroid hormone [72].

Iodine is stored in the thyroid gland and any excess consumed iodine is excreted in the urine [66]. Healthy adults can absorb more than 90% of the iodine they consume if required [66]. When the dietary intake of iodine is adequate, no more than 10% of absorbed iodine is taken up by the thyroid, but in chronic deficiency thyroid absorption can exceed 80% [66]. 

The primary dietary sources of iodine are dairy products, bread, seafood, meat and iodised salt [66,67,72]. However, within any population, the amount of iodine in its food sources varies greatly due to seasonal changes, plant and animal farming practices and processing techniques [66,72] and therefore iodine consumption varies considerably [67]. Iodine consumption also varies widely among individuals within a given population. For example, vegans are likely to have a diet deficient in iodine while those who eat kelp regularly may ingest excessive iodine [67]. 

Iodine deficiency was first shown to cause goitre (thyroid enlargement) in 1917 resulting in addition of iodine to table salt in Switzerland and the United Sates in the early 1920 to prevent the condition [66]. In 1980, the World Health Organization (WHO) estimated that 20%–60% of the world’s population was iodine deficient with the greatest prevalence in developing countries [66]. Studies conducted through 1970–1990 showed that supplementation in iodine deficient regions not only prevented goitre, but also eliminated other iodine deficiency disorders including cretinism, reduced infant mortality and improved cognitive function in the population [66]. Up until 1990, only Switzerland, some of the Scandinavian countries, Australia, the United States and Canada were routinely adding iodine to their table salt [66]. Since then, more than 70% of households globally use iodised salt thanks to the efforts of a coalition of international organizations including the International Council for the Control of Iodine Deficiency Disorders (ICCIDD), the World Health Organisation (WHO), the Micronutrient Initiative, UNICEF, national deficiency disorder committees and the salt industry [65]. However, iodine supplementation practices and dietary habits change in populations overtime making regular monitoring essential to identify both low and excessive iodine intakes [66].

Iodine status is determined by measuring the concentration of urinary iodine. Ninety percent of ingested iodine is assumed to be excreted in the urine so an individual’s iodine intake can be calculated based on the amount of urinary iodine excreted in a 24 h period. The WHO/UNICEF/ICCIDD recommended intake of 220–250 μg of iodine/day during pregnancy [68] and new recommendations from WHO suggest that a median urinary iodine concentration 250–500 μg/L indicates adequate iodine intake in pregnancy [71]. Based on this range, it appears that many pregnant women in Western Europe have inadequate intakes [71].

Currently, the WHO estimates that globally approximately 2 billion people have insufficient iodine intake [66]. Of the countries included in a 2008 survey by the ICCIDD, 11 had deficiency, 1 has moderate deficiency, 10 had mild deficiency, 20 were sufficient [73]. The top ten iodine deficient countries based on 2011 national median urine iodine concentration of <100 μg/L in school-aged children (*i.e.*, children with insufficient iodine intake) in consecutive order from worst to best were Pakistan, Ethopia, Sudan, Russian Federation, Afghanistan, Algeria, Angola, United Kingdom, Mozambique and Ghana [74]. Numerous studies in various countries have reported iodine deficiency in women of child bearing age, in pregnant women and in pregnant and lactating women even in areas where food fortification is undertaken (see Section 5.1 for details). 

As a developed country, the UK is an anomaly in the top ten iodine deficient countries mentioned above. Historically, iodine deficiency was widespread in Britain with high rates of goitre and even cretinism in some areas. Goitre was still present in Sheffield and South Wales until the 1960s. Goitre disappeared over the years owing to iodine supplementation in livestock to improve reproductive performance and lactation in the 1930s and iodophor disinfectants used for cleaning. Iodine intake increased for the next 30 years due to iodine contamination of milk through use of these cleaning agents. Also milk consumption increased due to free school milk and advertising by the Milk Marketing Board resulting in a three-fold increase in iodine intake between the 1950s and 1980s. Today, milk is the main source of iodine in the UK diet contributing 40% of the iodine intake [75]. However, milk consumption has decreased in recent years and iodophors are being replaced by other disinfectants [75]. At least one study has reported that low milk intake is linked to increased risk of low iodine status [76]. Contributing to the problem is increased consumption of organic milk over other sources since organic milk is 42.1% lower in iodine content than conventional milk [77]. Although iodised salt is available in the UK, only one brand with 0.6% market share is available, less than 20% of supermarket shoppers have iodised salt available for purchase, it is six times more expensive than non-iodised versions and 96% of UK pregnant women never or rarely eat iodised salt [78]. The UK National Diet and Nutrition Survey of 2000/2001 including adults aged 19 to 64 years reported a daily iodine intake of 215 μg/day in men and 159 μg/day in women where 12% of young women were consuming less than 70 μg/day [74]. Iodine intake had fallen since 1986/1987 and values reported in 2008/2009 showed a further fall [78].

The main health concern of mild iodine deficiency during pregnancy and while breastfeeding is its negative effect on the brain and nervous system development in the foetus and infant, in particular reduced intelligent quotient (IQ) [79,80,81,82,83]. Iodine deficiency during pregnancy leads to inadequate thyroid hormone production and hypothyroidism during pregnancy [67]. Thyroid hormone is required for normal neuronal migration, myelination, and synaptic transmission and plasticity during foetal and early postnatal life [68]. Hypothyroxinemia causes adverse effects on early foetal brain and nervous system development, can lead to irreversible foetal brain damage [72], and is the world’s most frequent cause of preventable mental retardation in later life [67]. The consequences depend on the timing and severity of the hypothyroxinemia [68]. Moderate-to-severe iodine deficiency during pregnancy also increases rates of spontaneous abortion, reduces birth weight, and increases infant mortality [84]. 

## 2. Evidence of the Potential Benefits of Maternal DHA Supplementation for Foetal/Infant Brain Health

### 2.1. Effects of Maternal DHA Supplementation on Maternal DHA Status

Numerous studies have confirmed that DHA supplementation either during pregnancy and/or while breast feeding can increase maternal stores of DHA in both her blood [18,85,86,87,88,89,90,91,92,93] and her breast milk [85,94,95]. A multi-centered, randomised, double-blind, placebo controlled trial including 311 pregnant women confirmed that daily supplementation with 500 mg DHA + 150 mg of the DHA precursor, eicosapentaenoic acid (EPA) from week 22 of gestation until delivery, significantly increased maternal plasma DHA (*p* < 0.001) relative to control [94]. A similarly designed single-centered study included 125 mothers of healthy full-term infants who daily consumed a placebo that did not contain any DHA or low dose tuna oil providing 300 mg DHA + 70 mg EPA or high dose tuna oil providing 600 mg DHA + 140 mg EPA (*n* = 40) from day 3 postpartum up to the end of week 12 postpartum [85]. DHA content increased relative to before treatment in both plasma and milk following tuna oil supplementation, but not after taking placebo. These studies [85,94] confirmed that DHA levels can be increased in the mother’s plasma and milk following supplementation with DHA from tuna oil*.*

### 2.2. Effects of Maternal DHA Supplementation on Foetal/Infant DHA Status

Many studies have reported enhanced DHA status in infants following maternal supplementation during pregnancy [18,86,89,90,91,96] or during lactation [92] or during both pregnancy and lactation [93,97]. A double-blind, randomised, placebo-controlled study including 83 women who received either 4 g of fish oil providing 2.24 g DHA and 1.12 g EPA or placebo per day from 20 weeks gestation until delivery reported the fatty acid composition of cord blood collected at the time of delivery in both groups [90]. The results showed that DHA was significantly higher (*p* < 0.001) in the cord blood of babies whose mothers were supplemented with fish oil than in those who took placebo. In addition, a significant increase (*p* < 0.001) in DHA in the mother’s blood directly correlated with a corresponding increase in the cord blood DHA indicative of infant DHA status. Another double-blind, placebo-controlled trial reported the effects of supplementing maternal diet for the first 12 weeks postpartum to achieve breast milk DHA concentrations ranging from 0.1% to 1.7% of the total fatty acids [92]. Analysis of 52 healthy term infant’s blood confirmed that increasing breast milk DHA levels caused a dose dependent increase in infant DHA status up to a maximum level where it then remained constant regardless of higher maternal DHA intake. When supplemented during pregnancy and lactation, a randomised, double-blind, placebo-controlled trial including 145 pregnant women provided 1.6 g EPA and 1.1 g DHA daily from the 25th gestational week through 3.5 months of breast feeding reported proportionally higher plasma DHA in infants from supplement mothers [93]. These study results confirm that maternal DHA supplementation during pregnancy and/or while breast-feeding improves foetal/infant DHA status.

### 2.3. Benefits to the Fetus/Infant/Child

#### 2.3.1. Observational Studies

**Figure 5:**
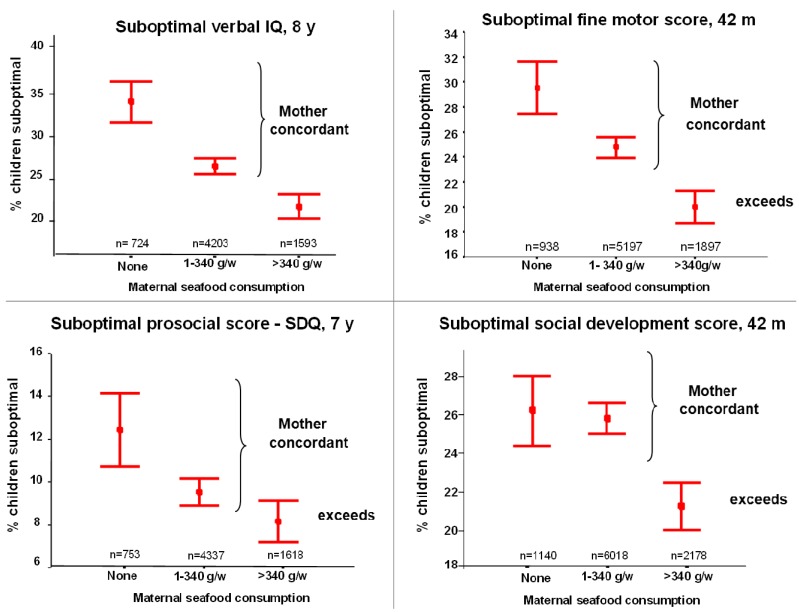
Offspring Outcomes in the ALSPAC Trial [98].

A flurry of observational research during the last decade has shown either the benefits that higher maternal and/or infant DHA status provide to the growing foetus and/or infant, or the risks associated with poor DHA status in either the mother or child to foetal/infant development and function. The pivotal study included data derived from the ALSPAC trial (Avon Longitudinal Study of Parents and Children) (Figure 5) [98]. It included 11,875 pregnant women living in Bristol, UK who completed a food frequency questionnaire to determine their seafood intake during pregnancy while the children were tested for development, behavior and mental function from age 6 months to 8 years. The women were divided into three categories based on seafood consumption: no seafood (12% of the women), some seafood (1–340 g per week, 65%) and greater than 340 g per week (23%). After results were adjusted to take into account 28 potential sources of interference, the verbal intelligence quotient (IQ) scores for children from mothers with no seafood intake were found to be 50% more likely to be in the group with the lowest IQ. Overall, low seafood intake during pregnancy was directly associated with suboptimal outcomes in the offspring for prosocial behavior, fine motor co-ordination, communication and social development.

Other studies have reported:

(1) Benefits of Maternal DHA Supplementation to the Foetus

- DHA status of preterm neonates is positively associated with measures of foetal growth including birth weight, head circumference and birth length [99]. In addition, as DHA increases, so does placenta weight [99].

(2) Benefits of Maternal DHA Supplementation to the Infant/Growing Child

- DHA status at birth is significantly and positively related to movement quality [100] and reduced problem behaviour at 7 years of age [101] visual acuity at age 6 months [102] and performance on behavioral tests including the Digit Span Forward Test and California Verbal Learning Test—Children’s Version at 11.3 years of age. Children with higher cord DHA, that is exposure to a higher amount of DHA during pregnancy, responded faster when making decisions that relied on memory than those with lower cord DHA. In addition, children with higher current DHA, that is higher routine intake of DHA, also responded faster when making decisions that relied on memory, than those with lower current DHA [103].
- Higher infant cord blood DHA concentration is directly associated with better mental and psychomotor development at 11 months of age [102] and visual system function in particular color detection in school aged children [104].
- Among children who were breast-fed for less than 6 months, maternal fish intake of greater than 2–3 times/week during pregnancy is associated with better scores on the McCarthy Scales of Children’s Abilities for verbal, perceptual-performance, quantitative, general cognitive, memory, and motor skills [105].
- Higher maternal plasma DHA during pregnancy is associated with more mature neonatal sleep-state, suggesting greater central nervous system maturity [106].
- Higher maternal DHA status at birth is associated with enhanced attention functioning during the second year of life [107].

All of these studies confirm that a higher prenatal and postnatal DHA concentration is more beneficial for infant visual, cognitive and motor development than a lower amount. 

#### 2.3.2. Intervention Trials

##### 2.3.2.1. During Pregnancy

**Figure 6:**
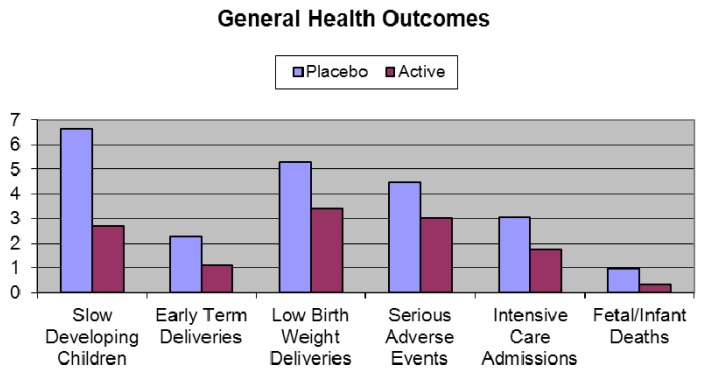
Infants general health outcomes in the DOMInO Trial [116].

The effects of DHA supplementation in pregnant women on foetal/infant outcomes has been evaluated in a number of randomized, double-blind, placebo-controlled trials providing 150–1200 mg/day DHA or up to 2.7 g total omega-3 LC-PUFAs/day. These have been systematically evaluated in two separate meta-analyses [108,109] and reported that omega-3 LC-PUFAs prolong gestation by 1.6 [108] and 2.6 [109] days, slightly increase birth weight by 47 g [108] and 54 g [109], and reduce the risk of preterm birth before 34 weeks gestation by 31% [108] in all pregnancies and by 61% [109] in high-risk pregnancies. In addition, excluding some minor discomfort including belching and unpleasant taste, no adverse effects were detected up to the highest dose of 2.7 g total omega-3 LC-PUFAs/day. Other studies have reported that:

- DHA reduces the incidence of premature delivery, increases birth weight, and gestation and may be useful to prolong gestational duration in some high-risk pregnancies [110].
- DHA increases infant birth weight and head circumference [111] and enhances growth (body length) through to 18 months in children from singleton pregnancies [112].
- Fish oil supplementation increases breast milk EPA and DHA content up to 6 weeks postpartum and these higher amounts are directly correlated with better Griffith’s developmental scores including hand and eye co-ordination in the infant at 1 year of age [94].
- DHA enhances visual acuity maturation in term infants, in particular in girls [2], attention and processing efficiency in infants [113], problem solving ability at 9 months of age [114] and hand/eye co-ordination at age 2.5 years [96].
- Higher foetal DHA exposure due to maternal supplementation results in better neurological outcome at 5.5 years of age [115]. The odds of children with maximal neurological optimality scores increases with every unit increment in cord blood DHA at delivery.
- The largest clinical study ever providing DHA to pregnant women was aptly named the DOMInO trial (DHA to Optimize Mother Infant Outcome) (Figure 6) [116]. The multicentered, randomised, double-blind, placebo-controlled clinical trial, conducted in 5 Australian maternity hospitals and supported by a grant from the Australian National Health and Medical Research Council included 2399 women with gestation of less than 21 weeks during singleton pregnancies and 726 of their infants. From twenty weeks until birth, the women took either three capsules providing 800 mg/day of DHA and 100 mg/day of EPA or three 500 mg/day vegetable oil capsules without DHA that matched the fatty acid composition of the average Australian diet. Cognitive and language development in the infants was assessed by the Bayley Scales of Infant and Toddler Development, Third Edition at 18 months of age. The primary outcome of cognitive and language development of infants in the DHA group did not differ from those in the control. However, major benefits were seen in disadvantaged slow developing children (those with an IQ of less than 85) where in all infants 6.64% in the placebo group were classed as “slow developers” compared to only 2.71% in the DHA group—a reduction of almost 60%. In boys, the reduction was even greater at 64%. Based on Australia’s birth rate this would represent 10,000 children per year no longer being classed as slow developers. For general health outcomes, DHA significantly reduced the incidence of low birth weight babies by 35% and the number of very early pre-term deliveries by more than 50% compared to the control. This represents a major public health benefit, in countries such as Australia for example, where there would be more than 3000 fewer preterm births per year if women were supplemented with DHA during pregnancy. Pre-term delivery and low birth weight are two of the major risk factors for ill health and poor mental development in children. Thirty-three percent less infants in the DHA group required admission to intensive care; there were two thirds less infant deaths in the DHA group and one third less infants in the DHA group experienced a serious adverse event relative to control. These findings were all highly significant and illustrate much better general health of the infants whose mothers were given DHA. There was no difference between groups for maternal hemorrhage, antenatal hospitalization, nose bleeds, vaginal blood loss, constipation, nausea or vomiting at 28 or 36 weeks gestation. However, more women in the DHA group reported eructations than the control group.

A one year follow up of this study reported no difference between the DHA and control group for IgE-mediated allergic disease or percentage of infants with food allergy [117]. However, more infants were sensitized to egg and had eczema in the control group than the fish oil group (15% of the control group were sensitive to eggs while only 9% of the DHA group was affected, and 12% of the control group had eczema while only 7% of the DHA group also suffered from the same condition). This difference corresponded to a higher DHA, EPA and total omega-3 fatty acid content in the cord blood of the DHA group *versus* the control group.

##### 2.3.2.2. During Lactation

To date, only a few studies have assessed the impact of maternal DHA supplementation solely during breast-feeding on infant development and function. Two hundred milligrams of DHA, for the first 4 months of breastfeeding, results in higher infant Bayley Psychomotor Development Index at 30 months of age [118] and better performance on tests of sustained attention. This suggests that DHA intake during early infancy confers long-term benefits on specific aspects of neurodevelopment [119].

##### 2.3.2.3. During Pregnancy and Lactation

A number of studies have reported benefits to the offspring following maternal DHA supplementation during both pregnancy and while breast feeding. One of the earliest randomized, double-blind, placebo-controlled trials included 84 children whose mothers took either 1183 mg/day DHA from cod liver oil or a corn oil placebo from week 18 of pregnancy until 3 months after delivery [120]. At age four years, the children were tested to measure IQ including problem solving and information processing abilities using the Kaufman Assessment Battery for Children designed for children from 2.5 to 12.5 years. The test is comprised of 4 scales: sequential processing, simultaneous processing, achievement (not included in this study), and nonverbal abilities. The sequential processing and simultaneous processing scales reflect the child’s style of problem solving and information processing and are combined to form a mental processing composite, which serves as the IQ. Those children who were born to DHA supplemented mothers scored higher on the IQ tests at 4 years of age as compared with children whose mothers had taken placebo. When retested at age 7 years, higher maternal DHA during pregnancy was associated with better sequential processing at 7 years of age [121]. 

## 3. Evidence of the Potential Benefits of Maternal Vitamin D Supplementation for Foetal/Infant Brain Health

Published placebo-controlled intervention trials studying the impact of vitamin D supplementation in mothers with low serum 25(OH)D are rare [45] because such trials are deemed unacceptable by ethics committees. Therefore, results of epidemiological studies provide most of the evidence suggesting the importance of vitamin D for foetal/infant brain health. 

### 3.1. Epidemiological Evidence

Vitamin D deficiency is common in pregnancy. A study in black and white pregnant women residing in the northern United States found that approximately 29% of black pregnant women and 5% of white pregnant women had vitamin D deficiency (serum 25(OH)D less than 37.5 nmol/L); whereas 54% of black women and 47% of white women had vitamin D insufficiency (defined as serum 25(OH)D levels 37.5 to 80 nmol/L) [122]. Recent studies in white pregnant women also show high prevalence of vitamin D deficiency in the UK [123] and Ireland [124]. Vitamin D deficiency has also been found in pregnant women residing in the southern United States [125] including a diverse group of African-American, Hispanic, and Caucasian pregnant women [126], in pregnant African-American adolescents [127], in pregnant Asian women [128], in veiled or dark-skinned pregnant women [129], in non-Western pregnant women in the Netherlands [130], and in pregnant women living in Belgium [131], Iran [132,133], India [134], Australia [135], Pakistan [136,137], Turkey [136], Somalia [136] and Oman [138]. Seasonal variation increases the risk of vitamin D deficiency in pregnancy, with greater prevalence of vitamin D deficiency during the winter months compared to the summer months [139]. Differences in latitude have also been shown to influence the concentration of vitamin D in a majority of pregnant women [140].

A recent review of studies linking maternal vitamin D status during pregnancy with maternal, foetal and postnatal outcomes supports a role of maternal vitamin D status, particularly early in pregnancy, in modulating the risk of pregnancy complications and in sustaining foetal growth, body composition, skeletal development, immune maturation and respiratory health [141]. Several studies have demonstrated an association between poor maternal vitamin D status and severe preeclampsia that can result in miscarriage [141]. Miscarriages can also result from an increased rate of bacterial vaginosis in the 1st trimester of pregnancy that is associated with low vitamin D status [49]. Maternal vitamin D status early in pregnancy was associated with risk of low birth weight and small-for-gestational age infants in one study, whereas another study found this relation only among white women [141]. Polymorphisms in the vitamin D receptor gene may contribute to vitamin D-related disparities in foetal growth [141]. Evidence from recent studies suggests an early prenatal influence of maternal vitamin D status on foetal skeletal development, with lasting postnatal effects [141]. In addition, one study has suggested that supplementation during pregnancy may be necessary to assure adequate concentration of vitamin D in breast milk during lactation [142]. Specifics of some studies are as follows:

#### 3.1.1. Studies Reporting Maternal Vitamin D Deficiency

A study measuring habitual micronutrient intakes at weeks 13, 25, 35 of pregnancy and 6 weeks postpartum using a prospective background information questionnaire, 4–7 days weighed food diary and postnatal questionnaire included 72 primiparous, Caucasian Londoners recruited at the study start with 42 completing the first, second, third trimester and postpartum study stages. Intakes of folate, iron, vitamin D, potassium, iodine and selenium were lower than UK recommendations during and after pregnancy (*p* < 0.05) [143].

- In a study completed by a coalition of scientists formed to optimize vitamin D fortification in the northern European countries, the average dietary intake of vitamin D in young women was only around 80 IU (2 μg) per day [144]. This falls short of even the most modest dietary recommendations.
- A cross-sectional study in Iran included 147 pregnant women whose serum status of vitamin D, A, and E were assessed at 5–9 months of pregnancy. The prevalence of vitamin D deficiency was 95.8% [133].
- The prevalence of vitamin D deficiency was determined in a diverse group of 559 women in South Carolina, USA at latitude 32°N. Mean age was 25.0 ± 5.4 (range 14–43) years; African American (48%), Hispanic (38%), Caucasian/Other (14%). Mean gestational age was 18.5 ± 8.4 (median 14.6, range 6.4–39.6) weeks. Vitamin D status was defined as 25(OH)D < 50 nmol/L deficiency; <80 nmol/L insufficiency. Forty-eight percent were vitamin D deficient, an additional 37% insufficient. The greatest degree was in the African American women (68% deficient; 94% insufficient) [125].
- Despite abundant sunshine and latitude consistent with year-long vitamin D synthesis, 65.5% of a largely low-risk antenatal population in rural Victoria, Australia had insufficient vitamin D. Over 5.0% of women had vitamin D levels that pose a significant neonatal and adult health risk [135].
- A cross-sectional study including 50 women in labour with a singleton term pregnancy in Pakistan measured vitamin D status in maternal blood before delivery and cord blood at delivery. Vitamin D sufficiency was noted in 11 (22%), insufficiency in 16 (32%), and deficiency in 23 (46%) of the 50 participants whereas sufficiency and deficiency, respectively, were noted in 6 (12%) and 44 (88%) of the newborns. There was a positive correlation between the vitamin D levels in maternal and cord blood (*r* = 0.03; *p* < 0.003). Maternal vitamin D levels were significantly affected by sunlight exposure (*p* < 0.007) and quality of diet (*p* < 0.01). The authors concluded that vitamin D deficiency is high among pregnant urban Pakistani women and their newborns and is a public health problem that needs urgent attention [137].

#### 3.1.2. Bone Health

- Doctors in Leicester City, UK reported that a significant number of south Asian mothers visiting their clinic had vitamin D deficiency at the end of pregnancy. A substantial number of their offspring had infantile and adolescent rickets including some with extremely severe bony deformities. In addition, there was an increase in late (5–10 days of age) and late-late (2–12 weeks of age) neonatal hypocalcaemia presenting predominantly with seizures, demonstrating the involvement of vitamin D in brain function [50].

#### 3.1.3. Pregnancy Maintenance

- A cohort study of 23,423 nulliparous pregnant women taking part in the Norwegian Mother and Child Cohort Study found a 27% reduction in risk of preeclampsia in women taking vitamin D supplements relative to those who did not take supplements [145]. However, because vitamin D intake is highly correlated with the intake of long chain *n*-3 fatty acids in the Norwegian diet, the authors cautioned that further research is needed to disentangle the separate effects of these nutrients.

#### 3.1.4. Brain Development

- Vitamin D’s nuclear hormone receptor regulates gene expression and nervous system development [54]. There is evidence that vitamin D during pregnancy is involved in foetal brain development and that maternal vitamin D deficiency during pregnancy can alter the structure and function of the brain resulting in life long behavioural changes in the offspring [146,147,148].
  ○ A pilot case-control study assessing the association between third trimester maternal serum 25(OH)D and the risk of schizophrenia included 26 cases and 51 controls. The results showed that 25(OH)D concentration varied by season and were lower in African American women as predicted. Within the African American mothers, a subgroup with markedly lower levels of 25(OH)D had a non-significant increase in schizophrenia [149].
  ○ A larger case-control study included 424 cases and matched control (sex and age) from the Danish Psychiatric Central Register. There was a significant seasonal variation in 25(OH)D and significantly lower 25(OH)D in the offspring of migrants. The risk of schizophrenia was significantly associated with neonatal 25(OH)D. Those with the lowest concentration had an increased risk of schizophrenia although the exposure risk was nonlinear (*i.e.*, higher than normal 25(OH)D was also associated with schizophrenia). Shifting all subjects to the optimal concentration of 25(OH)D could potentially avert 43.6% of schizophrenic cases in this group of patients. The authors concluded that prenatal vitamin D supplements in women at risk of deficiency could reduce the risk of schizophrenia in their offspring [149]. However, one should consider the complex nature of vitamin D effects since either deficiency or excess may be harmful [53].

### 3.2. Intervention Trials

#### 3.2.1. Studies Showing Correlation between Maternal and Foetal Vitamin D Status

- Five hundred and six pregnant women were given 400 IU (10μg) of vitamin D per day from about the 12th week of pregnancy until delivery [150]. A control group of 633 pregnant women was given a placebo. Maternal vitamin D was measured at the 24th and 34th weeks of pregnancy and at delivery and infant vitamin D was measured in umbilical blood at birth and on the sixth day following birth. Plasma concentrations of 25(OH)D, which showed a seasonal variation, was higher in mothers and infants in the treated group. Cord-blood 25(OH)D correlated with maternal values at delivery. A defect of dental enamel was found in a high proportion of infants (many of whom had suffered from hypocalcaemia) born to the control women. These results suggest that vitamin D supplementation during pregnancy would be beneficial for mothers, whose intake from diet and skin synthesis is appreciably less than 500 IU of vitamin D daily.

#### 3.2.2. Bone Health

- A prospective partially randomised study of vitamin D supplementation during pregnancy included Indian subjects (known to be vitamin D deficient) randomised in the second trimester to receive either one oral dose of 1500 μg vitamin D (group 1, *n* = 48) or two doses of 3000 μg vitamin D each in the second and third trimesters (group 2, *n* = 49) [151]. A control group included 43 non-supplemented mother-infant pairs under “usual care”. Median maternal 25(OH)D at term was higher in group 2 (58.7, interquartile range (IQR) 38.4–89.4 nmol/L) *vs.* group 1 (26.2, IQR 17.7–57.7 nmol/L) and Control group (39.2, IQR 21.2–73.4 nmol/L) (*p* = 0.000). Birth weight, length and head circumference were greater and the anterior fontanelle (soft spot on the head) was smaller in groups 1 and 2 (3.08 and 3.03 kg, 50.0 and 50.1 cm, 34.5 and 34.4 cm, 2.6 and 2.5 cm, respectively) *vs.* Control (2.77 kg, 49.4, 33.6, 3.3 cm; *p* = 0.000 for length, head circumference and fontanelle and *p* = 0.003 for weight). These differences were still evident at 9 months.

## 4. Evidence of the Potential Benefits of Maternal Folic Acid Supplementation for Foetal/Infant Brain Health

There are hundreds of published studies originating from various countries showing the benefit of folic acid supplementation before and during pregnancy to prevent neural tube defects. A 2010 Cochrane Review of evidence assessing folic acid supplements before conception and in early pregnancy (up to 12 weeks) for the prevention of birth defects confirmed that folic acid supplementation prevents the first and second time occurrence of neural tube defects and showed there is not enough evidence to determine if folic acid prevents other birth defects [152]. The review of five trials, involving 6105 women (1949 with a history of a pregnancy affected by a neural tube defect and 4156 with no history of neural tube defects), showed the protective effect of daily folic acid supplementation in doses ranging from 0.36 mg (360 µg) to 4 mg (4000 µg) a day, with and without other vitamins and minerals, before conception and up to 12 weeks of pregnancy, for preventing the recurrence of these diseases [152]. There were insufficient data to evaluate the effects on other outcomes such as cleft lip and palate. 

The impact of folic acid supplementation on prevention of neural tube defects has been extensively studied for decades resulting in individual reports too numerous to mention. Based on the early research, the US Preventive Services Task Force recommended in 1996 that all women planning a pregnancy or capable of conceiving take a supplement containing folic acid to reduce the risk of neural tube defects. A review of evidence accumulated since then and up to 2009 confirmed the previous scientific evidence supporting those recommendations [153]. The meta-analysis [153] included 1083 published articles of randomized, controlled trials, case-control studies and systematic reviews that reported an overall effect on reduction of neural tube defects or an effect on harms associated with folic acid containing supplements. Even though knowledge pertaining to the benefits of folic acid supplementation to prevent neural tube defects has been known for decades, a 2009 study reported that only 23%–38% of women met UK recommendations for folate through dietary sources [143]. The study measuring habitual micronutrient intakes at weeks 13, 25, 35 of pregnancy and 6 weeks postpartum using a prospective background information questionnaire, 4–7 days weighed food diary and postnatal questionnaire included 72 primiparous, Caucasian Londoners recruited at the study start with 42 completing the first, second, third trimester and postpartum study stages. Intakes of folate, iron, vitamin D, potassium, iodine and selenium were lower than UK recommendations during and after pregnancy (*p* < 0.05) [143]. 

## 5. Evidence of the Potential Benefits of Maternal Iodine Supplementation for Foetal/Infant Brain Health

### 5.1. Epidemiological Evidence

Numerous population studies from a variety of countries including China, Hong Kong, Iran, India, Kyrgyzstan and England have reported iodine deficiency in girls of child bearing age [76], in pregnant [154,155,156,157], and in pregnant and lactation women [158,159]. Some of these studies included regions where salt iodization is practiced, yet a significant proportion of pregnant and lactating women were still deficient [155,156,157,158,159,160,161]. A few examples of recent studies follow:

#### 5.1.1. Iodine Deficiency—Girls of Child Bearing Age

- A cross-sectional survey of iodine status systematically assessed in schoolgirls aged 14–15 years attending secondary school in nine UK centres included 810 participants provided 737 urine samples [162]. Data for dietary habits and iodine status were available for 664 participants. Urinary iodine measurements indicative of mild iodine deficiency were present in 51% (*n* = 379) of participants, moderate deficiency in 16% (*n* = 120), and severe deficiency in 1% (*n* = 8). Prevalence of iodine deficiency was highest in Belfast (85%, *n* = 135). Tap water iodine concentrations were low or undetectable and were not positively associated with urinary iodine concentrations. There were independent associations between low urinary iodine excretion and sampling in summer (*p* < 0·0001), UK geographical location (*p* < 0·0001), low intake of milk (*p* = 0·03), and high intake of eggs (*p* = 0·02). These results suggest that the UK population is iodine deficient. Since developing foetuses are the most susceptible to adverse effects of iodine deficiency and even mild perturbations of maternal and foetal thyroid function have an effect on neurodevelopment, these findings are of potential major public health importance. This study draws attention to an urgent need for a comprehensive investigation of UK iodine status and implementation of evidence-based recommendations for iodine supplementation [154].

#### 5.1.2. Iodine Deficiency—Pregnant Women

- A 2005 study including urban and rural sites from each of the 11 Chinese provinces concluded that effective iodised salt program has brought iodine sufficiency to most of China, but pregnant women in some areas may still risk deficiency and need further supplements [155].
- A 2008 State-wide survey in Rajasthan, an Indian State where the sale of non-iodised salt for human consumption was banned in 1992 reported that 41.9% of the households used salt containing insufficient levels of iodine, 23.0% used non-iodised salt and the median urine iodine concentration was 127 μg/L in pregnant women indicating iodine deficiency. These results indicate that household salt iodine content at its current mandated concentration does not supply sufficient iodine for pregnancy requirements [156].
- A national, Kyrgyzstan population-representative survey during autumn 2007 collected household salt and urine samples of school-age children and pregnant women for quantitative iodine measurements and measured maternal thyroid volume. Even though universal salt iodization was re-mandated in 2001 and 97.9% of salt samples were iodised, 39.5% had > or = 15 mg iodine/kg. The median urinary iodine concentration of pregnant women was only 111 μg/L and their thyroid volume increased with the duration of pregnancy. The iodine consumption among pregnant women from iodised salt did not assure their dietary requirements [157].
- A 2004 study to determine the prevalence of reduced iodine intake by measuring urinary iodide concentrations in pregnant and non-pregnant women from the north east of England included 227 women at 15 weeks gestation and 227 non-pregnant age matched controls. 3.5% of pregnant women had evidence of iodine deficiency, and 40% were borderline deficient [154].
- A case-control study completed in Surrey, UK included 100 women at 12 weeks gestation and 57 women of childbearing age as a control. Based on urine analysis, the pregnant women were mild to moderately iodine deficient. Seventy-five percent of participants took a nutritional supplement but only 42% took a supplement containing iodine. Significantly lower iodine levels were found in those who did not consume milk daily [163].

#### 5.1.3. Iodine Deficiency—Pregnant or Lactating Women

- A 2007 review of cross-sectional and prospective studies to describe the iodine nutrition of pregnant and lactating women in Hong Kong, where intake is of borderline sufficiency revealed an increase in the urinary iodine concentration as pregnancy advances. A significant percentage of women had a sub-normal serum thyroid hormone concentration at full term. Although iodine is concentrated by the mammary gland, 19% of all mothers had low iodine concentrations in their breast milk. The moderate correlation between the concentrations of iodine in breast milk and urine suggests that an adequate maternal urinary iodine concentration cannot reliably indicate that an infant is getting enough iodine in breast milk. Therefore, some breast-fed infants may still be at risk of low iodine intake, and additional iodine supplements, other than salt iodisation, would be warranted in this population [158].
- A cross-sectional study conducted in Iran between 1996 and 1998 in 403 pregnant women and a study of 100 lactating women conducted in 2003 included evaluated thyroid size, and both urinary and breast milk iodine concentrations. When data were combined for the cities of Ilam, Isfahan and Tehran, where women have an adequate or more than adequate median urinary iodine concentration, 51% of pregnant women had a urinary iodine concentration less than that recommended during pregnancy. The mean urinary iodine concentration in lactating women was 250 μg/L, and 16% of women had a urinary iodine concentration <100 μg/L. Grade 1 goitre was present in 8% of lactating women, and another 8% had grade 2 goitre [159].
- A study including 433 pregnant and 95 non-pregnant women in Tayside, Scotland, mean gestational age at recruitment of 11.5 weeks measured urinary iodine and a range of thyroid hormones. Even though iodised salt was available in the area, only 30% of women consumed it and the iodine intake of these women had not increased to meet the higher requirements of pregnancy (~250 µg/day). Indeed, the urinary iodine was the same in pregnant and non-pregnant women. Approximately 40% of the pregnant women from this area of the UK had urinary iodine excretion below those corresponding to half the recommended intake [160]. The ensuing failure to increase their T4 during the 1st trimester of pregnancy may well have adverse effects on the progeny’s neurodevelopment [77].

#### 5.1.4. Studies Showing Maternal Thyroid Deficiency Impacts Brain Development of Her Child

- Serum samples collected from 25,216 pregnant women between January 1987 and March 1990 were tested for thyrotropin to recruit 47 women with serum thyrotropin concentration at or above the 99.7th percentile of the values for all the pregnant women, 15 women with values between the 98th and 99.6th percentiles, inclusive, in combination with low thyroxine levels, and 124 matched women with normal values. Their seven-to-nine-year-old children, none of whom had hypothyroidism as newborns, underwent 15 tests relating to intelligence, attention, language, reading ability, school performance, and visual-motor performance. The children of the 62 women with high serum thyrotropin concentrations performed slightly less well on all 15 tests. Their full-scale IQ scores on the Wechsler Intelligence Scale for Children, third edition, averaged 4 points lower than those of the children of the 124 matched control women (*p* = 0.06); 15% had scores of 85 or less, as compared with 5% of the matched control children. Of the 62 women with thyroid deficiency, 48 were not treated for the condition during the pregnancy under study. The full-scale IQ scores of their children averaged 7 points lower than those of the 124 matched control children (*p* = 0.005); 19% had scores of 85 or less. Eleven years after the pregnancy under study, 64% of the untreated women and 4% of the matched control women had confirmed hypothyroidism. Although this study did not include testing for iodine status during pregnancy, it does show that undiagnosed hypothyroidism in pregnant women may adversely affect their offspring [161].

#### 5.1.5. Studies Showing Maternal Iodine Status Impacts Brain Development of Her Child

Many studies have reported an association between severe iodine deficiency and poor mental development as illustrated in a meta-analysis of studies conducted on children born and raised in areas before and after iodine food fortification [164]. However, recently studies have emerged confirming the link between moderate or mild iodine deficiency during pregnancy and offspring intellectual capacity. Some of these studies are summarized below:

##### 5.1.5.1. Severe Deficiency

- A meta-analysis of 37 studies including 12,291 sixteen year olds born and raised in China before and after iodine food fortification compared to those living in naturally iodine sufficient locations (IS) with those in severely iodine deficient (ID) areas, or children in ID areas born before and after the introduction of iodine supplementation. IQ was measured using Binet or Raven Scales. There was a 12.45, 12.3 and 4.8 increase in IQ points respectively, for the children living in IS communities compared with:
  ○ Those living in ID areas with no iodine supplementation;
  ○ With inadequate iodine supplementation;
  ○ Or children who had received iodine during their mothers’ pregnancy and after birth.

Compared with that of children whose mothers were persistently exposed to ID, the combined total effect of iodine supplementation during pregnancy was an increase of 8.7 IQ points. Furthermore, there was an increase of 12 IQ points for children born more than 3.5 years after iodine supplementation program was introduced. The level of iodine nutrition plays a crucial role in the intellectual development of children. The intelligence damage of children exposed to severe ID was profound, demonstrated by 12.45 IQ points loss that recovered 8.7 IQ points with iodine supplementation or IS before and during pregnancy. Results of this study showed that iodine supplementation before and during pregnancy to women living in severe ID areas could prevent their children from intelligence deficit. This effect becomes evident in children born 3.5 years after the iodine supplementation program was introduced [164].

##### 5.1.5.2. Mild to Moderate Deficiency

- Iodine status was investigated in 1,000 women of the Avon Longitudinal Study of Parents and Children (ALSPAC) cohort who were recruited in the 1990s. Iodine concentration (and creatinine to adjust for urine volume) was measured in urine samples from pregnant women of median gestational age 13 weeks [83]. Women were grouped as iodine-deficient or sufficient according to WHO criteria. The relationships between maternal iodine status and child’s IQ at age 8 (Wechsler Intelligence Scale for Children), reading ability at age 9 (Neale Analysis of Reading Ability), and Key Stage 2 scores at age 11 were analysed using logistic regression. The group was mildly-to-moderately iodine deficient and 61% of women were classed as iodine deficient when using the creatinine-adjusted data. The children of women deficient in iodine were more likely to have a total IQ score below the 25th percentile (unadjusted OR = 1.42, 95% CI 1.05–1.94) after adjusting for mother’s parenting score, home score, family adversity during pregnancy, life-event score, dietary intake of *n*-3 fatty acids and iron, gender, ethnicity, maternal age, smoking, alcohol intake, parity, breastfeeding, partner at birth, parental education, housing status, crowding and use of iron, fish oil and vitamin/mineral supplements. The level of maternal iodine appeared sufficient to affect brain development in the offspring as shown by:
  ○ Significantly lower total IQ at age 8;
  ○ Significantly lower reading accuracy at age 9;
  ○ Poorer school performance at age 11, including significantly poorer in mathematics.

These results suggest the importance of achieving adequate iodine status during pregnancy and highlight the possibility that iodine deficiency can pose a risk to the developing infant, even in a country considered to be iodine replete. 

### 5.2. Intervention Trials

To date, most trials involving iodine supplementation during pregnancy have reported effects on maternal and/or infant thyroid function and have not specifically measured indicators of brain development and function [66]. Even so, authors of these studies have argued that even mild-to-moderate iodine deficiency in pregnancy similar to that seen presently in Europe, may negatively affect cognitive function in the offspring [66]. Of those studies that have assessed offspring brain development and/or function, some [165], but not all [166] have reported improvements following supplementation. A study including 133 women who received 300 μg/day of potassium iodide during the first trimester of pregnancy and 61 women who received no iodine evaluated the psychobiological development of their infants aged 3 to 18 months. The neuropsychological status of the children was evaluated with the Bayley Scales of Infant Development, and measurements were made of TSH, free T3, free T4, and urinary iodine. Those children whose mothers received iodine supplementation had more favourable psychometric outcomes including higher scores on the Psychomotor Development Index (*p* = 0.02) and the Behaviour Rating Scale than those of the non-supplemented group. This study showed that dietary iodine supplementation during pregnancy had no harmful effect on the neurodevelopment of the children and was instead beneficial [165]. However, in a double blind controlled trial in five villages in Papua New Guinea, several measures of motor and cognitive function showed no significant differences at either age 11 or 15 years between those children whose mothers had received supplementary iodine during pregnancy and the control children whose mothers had received the placebo [166].

## 6. Safe Intake Recommendations

### 6.1. DHA

Scientific data collected prior to 2008 established that dietary fat intake in pregnant women affects pregnancy outcome and fat intake during pregnancy and while breast-feeding impacts the growth, development and health of their offspring. Given the importance of this issue for public health, the European Commission charged the European research project, PeriLip (*Influence of Dietary Fatty Acids on the Pathophysiology of Intrauterine Foetal Growth and Neonatal Development*) and EARNEST (*Project Coordinating Committee of the Early Nutrition Programming project*), with the task of developing recommendations on dietary fat intake in pregnancy and lactation, based on scientific evidence [167,168]. These groups included representatives of the:

- Child Health Foundation;
- Diabetic Pregnancy Study Group;
- European Association of Perinatal Medicine;
- European Society for Clinical Nutrition and Metabolism;
- European Society for Pediatric Gastroenterology;
- Hepatology and Nutrition;
- Committee on Nutrition;
- International Federation of Placenta Associations;
- International Society for the Study of Fatty Acids and Lipids.

This authoritative body of experts undertook an extensive review of current scientific evidence to develop recommendations for dietary fat, fatty acid and antioxidant intake during pregnancy and lactation. Their review included omega-3 LC-PUFA intakes for women with low and high risk pregnancies, intakes during lactation and their effects on human milk composition and infantile outcome, effects of antioxidant intakes in pregnant and lactating women and toxicological evaluations on sea fish consumption in women of childbearing age. 

This review established that:

- Fat, as a proportion of total energy needs should be the same in pregnant and lactating women as recommended for the normal population.
- Pregnant and lactating women require at least 200 mg of DHA per day. Numerous populations studies throughout westernized countries have confirmed that our intake is much lower than this, with a mean of about 150 mg per day [169].
- Maternal intake of fish, fish oils or omega-3 LC-PUFAs result in a slightly longer duration of gestation, a somewhat higher birth weight and a reduced risk of early preterm delivery.
- The foetus and neonate must receive sufficient LC-PUFA to support optimal visual and cognitive development.
- Breast feeding is endorsed as the preferred method of feeding to supply LC-PUFAs to the growing infant for the first 6 months of life. Dietary LC-PUFA supply should also continue after that time, but currently there is insufficient data to provide specific recommendations.
- Dietary intakes up to 1 g DHA/day or 2.7 g EPA + DHA/day have been used in clinical trials without occurrence of significant adverse effects.

There is no clinical evidence to support a safety concerns for DHA supplementation during lactation. Intake of 2 g/day of combined EPA and DHA is similar to that seen in large sectors of the Japanese population and well below that of Greenland Inuit, both of whom suffer no ill effects from this routine consumption throughout all phases of their lives including breast feeding [170]. 

### 6.2. Vitamin D

Currently there are no consistent recommendations amongst or even within countries for vitamin D intake during pregnancy and lactation. Independent researchers have recommended up to 100 μg (4000 IU) daily to increase maternal and neonatal vitamin D status to optimal levels [171]. In 2011, the US Endocrine Task Force on vitamin D stated that 15 μg (600 IU) daily may not be enough to correct vitamin D deficiency in pregnant and lactating women. Their recommendation was 37.5–50 μg 1500–2000 IU) per day in pregnant and lactating women with vitamin D deficiency [172]. Table 1 includes recommendations from various health agencies and governments around the world.

**Table 1:** Vitamin D intake recommendations during pregnancy and lactation.

Agency/Government	Recommendation
UK—for the elderly, pregnant & lactating women [173]	Dietary Reference Values 10 µg/day (400 IU/day)
UK Department of Health 2007—for pregnant and lactating women [45]	10 µg/day (400 IU/day)
UK National Institute of Health and Clinical Excellence Guideline Review Panel 2007 [45]	All women should be informed about the importance for their own and their baby’s health of maintaining adequate vitamin D stores during pregnancy and whilst breast feeding and may choose to take 400 IU/day.
Canadian Paediatric Society [45]	50 μg/day (2000 IU/day) throughout pregnancy
Federal Department of Health Canada [45]	5 μg/day (200 IU/day) for pregnant and breast-feeding women
European Commission [45]	10 μg/day (400 IU/day) during pregnancy
World Health Organisation 2004 [45]	5 μg/day (200 IU/day) during pregnancy
The Institute of Medicine US 2010 [172,174]	15 μg/day (600 IU/day) in pregnant and lactating women
US Endocrine Task Force on Vitamin D 2011 [174]	37.5–50 μg/day (1500–2000 IU) in pregnant and lactating women with vitamin D deficiency.

A recent Cochrane Review assessed the effects and safety of vitamin D supplementation in pregnancy and examined whether supplementation with vitamin D alone or in combination with calcium and other vitamins and minerals given to women during pregnancy could safely improve pregnancy outcomes [175]. The review included five trials involving 623 women comparing the effects of vitamin D alone *versus* no supplementation/placebo and one trial with 400 women comparing the effects of vitamin D and calcium *versus* no supplementation. Data from four trials involving 414 women consistently showed that women who received vitamin D supplements during pregnancy had higher concentrations of vitamin D in serum at term than those women who received no intervention or a placebo; however the magnitude of the response was highly heterogenous. Data from three trials suggested that vitamin D supplemented women had babies with birth weights below 2500 g less frequently than those women receiving no treatment or placebo. Women with pre-eclampsia who received 1200 IU vitamin D along with 375 mg of elemental calcium per day were as likely to develop pre-eclampsia as women who received no supplementation. There were no significant differences in adverse side effects including nephritic syndrome, stillbirths or neonatal deaths between women who received vitamin D supplements relative to women who received no treatment or placebo. The authors concluded that vitamin D supplementation in a single or continued dose during pregnancy increases serum vitamin D concentrations. However, due to the small number of high quality studies currently reported, the clinical significance of this finding and the potential safe use of this intervention as part of routine antenatal care are yet to be determined through rigorous randomised trials. 

There has been little toxicity reported in adults taking doses of vitamin D as high as 10,000 IU/day (250 µg/day) of vitamin D [176,177,178] although toxicity becomes generally present at 20,000 IU/day (500 µg/day). Recently, a randomized, controlled trial, including 350 women with a singleton pregnancy at 12 to 16 weeks’ gestation supplemented with 400 (10 μg), 2000 (50 μg), or 4000 IU (100 μg) of vitamin D per day until delivery. The primary outcome was maternal/neonatal circulating 25(OH)D concentration at delivery, with secondary outcomes of a 25(OH)D concentration of 80 nmol/L or greater achieved and the 25(OH)D concentration required to achieve maximal 1,25-dihydroxyvitamin D(3) production. There were no differences between groups on any safety measure. Not a single adverse event was attributed to vitamin D supplementation or circulating 25(OH)D levels. The authors concluded that vitamin D supplementation of 4000 IU/day for pregnant women is safe and most effective in achieving sufficiency in all women and their neonates regardless of race, whereas the current estimated average requirement is comparatively ineffective at achieving adequate circulating 25(OH)D concentrations, especially in African Americans [179].

### 6.3. Folic Acid

The recommended dietary allowance for women of childbearing age is 400 μg/day of folic acid according to the Institute of Medicine [58]. These recommendations are based on the amount of dietary folate equivalents needed to maintain normal red blood cell concentration. In addition to this dietary recommendation, all women who may become pregnant should take a multivitamin containing 400 μg/day of folic acid to reduce the risk of neural tube defects. These recommendations are recognized and endorsed around the world [63,64,180,181,182,183,184]. Some countries provide additional recommendations such as those in New Zealand where women at low risk of a neural tube defect affected pregnancy who plan to become pregnant, are recommended to take a 800 µg of folic acid daily for at least four weeks prior to conception and for 12 weeks after conceiving to reduce the risk of neural tube defects [64]. 

Folate intake from food is not associated with any health risk. The risk of toxicity from folic acid intake from supplements and/or fortified foods is also low [185]. It is a water soluble vitamin, so any excess intake is usually lost in the urine. There is some evidence that high levels of folic acid can provoke seizures in patients taking anti-convulsant medications [186] and recommendations are that anyone taking such medications should consult with a medical doctor before taking a folic acid supplement. 

A 2009 meta-analysis including 1083 published articles of randomized, controlled trials, case-control studies and systematic reviews assessing the harms associated with folic acid containing supplements did not find any association of folic acid supplementation with either twin pregnancy or masking vitamin B12 deficiency (both concerns previously raised in the literature). One fairly well designed study suggested that confounding by infertility treatment explains previously reported associations of folic acid and twin pregnancy. The retrospective cohort study examined the association between risk for twining in 176,042 women who gave birth in Norway between December 1998 and December 2001 and their history of multivitamin or folic acid use before or during pregnancy. After adjusting for age, parity, underreporting of folic acid use and *in vitro* fertilization, the OR for twin delivery after preconceptional supplementation was 1.02 (CI, 0.85 to 1.24) and was about the same as for women who did not take folic acid [153]. 

The Institute of Medicine has established a tolerable upper intake level (UL) for folate from fortified foods or supplements (*i.e.*, folic acid) for ages one and above. Intakes above this level increase the risk of adverse health effects. In adults, supplemental folic acid should not exceed the UL to prevent folic acid from triggering symptoms of vitamin B_12_ deficiency [58]. It is important to recognize that the UL refers to the amount of synthetic folate *(i.e.*, folic acid) being consumed per day from fortified foods and/or supplements. There is no health risk, and no UL, for natural sources of folate found in food. Table 2 lists the UL for folate, in micrograms (μg), for women of child bearing age.

**Table 2:** Tolerable upper intake levels for folate in women [58].

Age (Years)	Females (μg/day)	During Pregnancy (μg/day)	During Lactation (μg/day)
9–13	600	N/A	N/A
14–18	600	800	800
>19	1000	1000	1000

### 6.4. Iodine

Recently WHO/UNICEF/ICCIDD increased the Recommended Nutrient Intake (RNI) for iodine during pregnancy and lactation to 250 μg/day [66,187]. The RNI is the intake estimated to cover the needs of “nearly all” healthy individuals in the specified life stage. In addition, women should take iodine supplements (in the recommended dose for pregnancy) from the point of planning pregnancy through the full duration of pregnancy and breast feeding [72]. Risks associated with iodine supplementation in the recommended doses are low since only a small amount of iodine can be stored in the body and any excess is excreted [72]. Women with pre-existing thyroid conditions should seek advice from their medical practitioner before taking an iodine supplement [72]. Table 3 lists the intake recommendations from various health agencies and governments around the world.

**Table 3:** Recommended Iodine Intake.

Agency/Government	Recommendation
The Australian National Health and Medical Research Council [188]	220 μg/day for pregnant women and 270 μg/day for breast feeding women
New Zealand Ministry of Health [188]	220 μg/day for pregnant women and 270 μg/day for breast feeding women
US Food and Nutrition Board of the Institute of Medicine [66,189]	220 μg/day for pregnant women and 290 μg/day for lactating women
The American Thyroid Association [165]	150 μg/day during pregnancy [67] and lactation, and that vitamins for prenatal use or use during pregnancy should be enriched with 150 μg/day of iodine

In 2002, the EU Scientific Committee on Food completed a thorough review of the existing safety data pertaining to iodine intake and reported the UL of Iodine to be 1700 and 1800 μg/day for adults. The UL of 600 μg/day was considered to be acceptable for pregnant and lactating women based on evidence of lack of adverse effects at exposures significantly in excess of this level [190].

## 7. Conclusions

A substantial amount of scientific research highlights the critical role that maternal nutrient intake during pregnancy and lactation plays to ensure normal offspring function. Many dietary nutrients are required for growth and development of the brain and central nervous system. However, common dietary deficiencies within many populations of particular nutrients including DHA, vitamin D, folic acid, and iodine, that all play critical roles at various developmental stages, have been shown to contribute to functional abnormalities, many of which have lasting effects. 

Adequate maternal intake of DHA during pregnancy and lactation is necessary for proper cell membrane formation in the brain and central nervous system and to ensure healthy foetal growth including birth weight, head circumference and birth length. Intervention trials have reported that DHA supplementation can prolong gestation in high risk pregnancies, increase birth weight, head circumference and birth length, enhance infant development including hand and eye co-ordination up to 2.5 years of age, visual acuity, attention processing efficiency, better neurological outcomes up to 5.5 years, and problem solving ability, information processing and IQ up to age 7 years. In addition, it can reduce the incidence of “slow developers”.

Vitamin D is involved in the regulation of cellular differentiation and apoptosis thereby exerting effects on foetal skeletal growth, development of the immune system and the brain. Preclinical studies in offspring born to vitamin D deficient mothers have reported gross morphological changes in brain structure that persist into adulthood resulting in impaired attention processing, sensitivity to agents that induce psychosis and abnormal movement patterns. Epidemiological studies have linked low maternal vitamin D status to severe preeclampsia resulting in miscarriage, risk of low birth weight and small-for-gestational age infants, neonatal hypocalcaemia with seizures and possible involvement in the development of schizophrenia. 

Folic acid is necessary for cell division, synthesis of amino acids and nucleic acids and ultimately for normal development of the foetal spine, brain and skull in particular during the first four weeks of pregnancy to prevent neural tube defects including spina bifida and anencephaly. The impact of folic acid supplementation on prevention of neural tube defects has been extensively studied for decades resulting in the requirement for folic acid supplementation in women of child bearing age and during pregnancy becoming well established and internationally recognized.

Iodine is essential for normal thyroid hormone production needed for normal brain and nervous system development during gestation. Epidemiological studies report that severe maternal iodine deficiency results in poor mental development of offspring including significantly reduced IQ while even mild to moderate deficiency negatively impacts IQ, reading accuracy and school performance. Intervention trials assessing offspring cognitive and motor function following maternal iodine supplementation during pregnancy are scarce and have reported significant enhancements up to 18 months, but no significant improvements relative to control at age 11 or 15 years. 

Maternal supplementation with DHA, vitamin D, folic acid and iodine within recommended safe intake quantities in a large segment of the population that is currently deficient in these nutrients, could significantly prevent many brain and central nervous system malfunctions and even enhance brain development and function in future generations. 
